# Plasticized Polymer Blend Electrolyte Based on Chitosan for Energy Storage Application: Structural, Circuit Modeling, Morphological and Electrochemical Properties

**DOI:** 10.3390/polym13081233

**Published:** 2021-04-11

**Authors:** M. H. Hamsan, Muaffaq M. Nofal, Shujahadeen B. Aziz, M. A. Brza, Elham M. A. Dannoun, Ary R. Murad, M. F. Z. Kadir, S. K. Muzakir

**Affiliations:** 1 Centre for Foundation Studies in Science, University of Malaya, Kuala Lumpur 50603, Malaysia; hafizhamsan93@gmail.com (M.H.H.); mfzkadir@um.edu.my (M.F.Z.K.); 2 Department of Mathematics and General Sciences, Prince Sultan University, P. O. Box 66833, Riyadh 11586, Saudi Arabia; muaffaqnofal@gmail.com; 3 HameedMajid Advanced Polymeric Materials Research Lab., Department of Physics, College of Science, University of Sulaimani, Qlyasan Street, Sulaimani 46001, Iraq; mohamad.brza@gmail.com; 4 Department of Civil Engineering, College of Engineering, Komar University of Science and Technology, Sulaimani 46001, Iraq; 5 Associate Director of General Science Department, Woman Campus, Prince Sultan University, P. O. Box 66833, Riyadh 11586, Saudi Arabia; elhamdannoun1977@gmail.com; 6 Department of Pharmaceutical Chemistry, College of Medical and Applied Sciences, Charmo University, Chamchamal 46023, Iraq; ary.murad@charmouniversity.org; 7 Material Technology Program, Faculty of Industrial Sciences and Technology, Universiti Malaysia Pahang, Pekan 26300, Malaysia; saifful@ump.edu.my

**Keywords:** plasticized polymer electrolyte, electrochemical impedance spectroscopy, XRD and morphology analyses, TNM and LSV measurements, CV study, EDLC device

## Abstract

Chitosan (CS)-dextran (DN) biopolymer electrolytes doped with ammonium iodide (NH_4_I) and plasticized with glycerol (GL), then dispersed with Zn(II)-metal complex were fabricated for energy device application. The CS:DN:NH_4_I:Zn(II)-complex was plasticized with various amounts of GL and the impact of used metal complex and GL on the properties of the formed electrolyte were investigated.The electrochemical impedance spectroscopy (EIS) measurements have shown that the highest conductivity for the plasticized system was 3.44 × 10^−4^ S/cm. From the x-ray diffraction (XRD) measurements, the plasticized electrolyte with minimum degree of crystallinity has shown the maximum conductivity. The effect of (GL) plasticizer on the film morphology was studied using FESEM. It has been confirmed via transference number analysis (TNM) that the transport mechanism in the prepared electrolyte is predominantly ionic in nature with a high transference number of ion (*t_i_*)of 0.983. From a linear sweep voltammetry (LSV) study, the electrolyte was found to be electrochemically constant as the voltage sweeps linearly up to 1.25 V. The cyclic voltammetry (CV) curve covered most of the area of the current–potential plot with no redox peaks and the sweep rate was found to be affecting the capacitance. The electric double-layer capacitor (EDLC) has shown a great performance of specific capacitance (108.3 F/g), *ESR*(47.8 ohm), energy density (12.2 W/kg) and power density (1743.4 W/kg) for complete 100 cycles at a current density of 0.5 mA cm^−2^.

## 1. Introduction

Polymer materials provide many options in the field of industry compared to other types of materials. In recent decades, the application of polymer electrolytes (PEs) using H^+^ ion as a charge carrier hasbeen extensively investigated in the field of energy storage devices (ESDs) [1,2]. Solid polymer electrolytes (SPEs) are one of the types of PEs that have been widely studied. SPEs can be prepared by hosting inorganic salts such as Li^+^ and Zn^2+^ in polymers that comprise heteroatoms such as sulfur, oxygen and nitrogen [3,4,5]. Polymer electrolytes have a broad range of applications in ESDs. Supercapacitors (SCs) are well recognized and have progressed in the field of energy storage technology. Based on an charge storage mechanism, SCs are generally classified into two types, which are the electric double-layer capacitor (EDLC) and pseudocapacitor (PC) [6,7]. The SCs have expressively aged over the past few years and have displayed the abilityto offer developments forthe ESDs. SCs are a promising next-generation energy storage technology.

Typically, EDLCs use carbon type materials [8], for example carbon nanotubes, graphene, carbon aerogel and activated carbon. In recent times, many researchers have beenpassionate about preparing innovative electrode materials for EDLC applications. For instance, carbon nano-onions (CNO) and boron-doped diamond (BDD) have been employed as an electrode material for SCs [9,10]. Nevertheless, these electrode materials have high processing costs, which limit its viable applications and cause it to have a less active surface area. Activated carbon-based electrode materials are preferably used in EDLCs due to their excellent specific surface area, thermal stability, electronic conductivity and good electrochemical stability. Activated carbon is easy to handle as well due to it beingcompatible with various binders, solvents and electronic conductors [11].

Blending two or more natural polymers is one of the effective ways to produce a biodegradable polymers host with high ionic conductivity, excellent thermal properties, good mechanical strength and low toxicity [12]. Biodegradable polymers such as chitosan (CS), starch, cellulose, dextran (DN), gelatin and carrageenan are also blended with non-biodegradable polymers such as polyvinyl pyrrolidone (PVP) and polymethyl methacrylate (PMMA) to reduce the cost as well as the plastic waste pollution. Jothi et al. [13] blended 80 wt.% corn starch with 20 wt.% PVP and achieved a high amorphous polymer host. DN is a natural polymer which is made from the fermentation of leuconostocmesenteroides bacteria [14]. DN as a drug carrier has been widely used in the field of the medical industry [15]. The polymer backbones of DN composed of polysaccharide which are linked via 1,6-α-d-glucopyranosidic units. Owing to the present of oxygen in its functional groups, DN is considered as a good candidate for ionic conduction in the area of polymer electrolytes [16,17]. CS is another class of biodegradable polymer that has been used in this work. CS is prepared by treating the chitin shells of shrimp and other crustaceans with an alkaline substance, such as sodium hydroxide. CS has a numeral of marketable and promising biomedical uses. Thus, CS is a derivative biodegradable polymer, which is obtained from the natural resources. The structure of CS is composed of β-1,4-linked polymer of 2-amino-2-deoxy-d-glucose-(d-glucosamine), which enables the positive charge from amine groups to migrate between electrodes [18,19,20].

For the preparation of electrolytes based on biodegradable polymer materials, the addition of zinc metal complexes (Zn(II)-complexes) into the polymer can offer anamorphous nature to the polymer structure. This can be considered as a good candidate filler to enhance the ionic conduction mechanism within the electrolyte. Asnawi and co-workers documented that the efficiency of EDLC device was enhanced when the Zn(II)-complexes was incorporated into theCS polymer electrolyte. It was noted that the addition of the metal complex improved the amorphous structure of the polymer. Brza and co-workers reported that, by adding cupper metal complexes (Cu(II)-complexes) into polyvinyl alcohol (PVA) polymer electrolyte, the performance of the EDLC device as well as the amorphous phase were enhanced [1,21].

The lattice energy of salts is influenced by the size of ions. Low lattice energy can be acquired when large size of anion and small size of cation are subjected. It is noteworthy that ammonium salts have high ionic conductivity. The conductivity of ions can be improved by hosting ammonium iodide (NH_4_I) into the polymer electrolyte, as well as utilizing a plasticizer such as GL, polyethylene glycol (PEG), ethylene carbonate (EC), and polyethylene carbonate (PEC), which reduce the viscosity of the polymer and increase the mobility of ions [22,23,24]. Moreover, owing to the three hydroxyl groups in the glycerol’s structure, this type of plasticizer is a good choice to improve the conductivity.

The GL has been reported to be compatible with most natural polymers. Andrade et al. [25] added GL to pectin-lithium perchlorate (LiClO_4_) electrolyte and achieved a conductivity of 4.7 × 10^−4^ S/cm.

The energy production and storage devices have caused serious issues, especially their environmental impacts and global warming issues [26,27]. Furthermore, the related electronic waste has beenfound to be fatal for many living species. These factors have encouraged many research groups to focus on replacing non-renewable petroleum based materials with biodegradable natural polymers like CS [26,27,28]. The current study emphasizes the preparation and studies the biodegradable and eco-friendly polymer electrolytessuch as CS and DN, as an attempt to reduce electronic waste. This study is considered as a part of hugeattempt and large research conducted by scientistsaiming at commercializing the biodegradable polymer electrolytes in energy devices like supercapacitors. Consequently, this research is exploring the possibility and suitability of using biodegradable polymers in energy devices, which can have both environmental and economic benefits. In this work, CS and DN are chosen as the polymer hosts with ammonium iodide as the ionic source. The highest conducting electrolyte is used to fabricate the EDLC device.

## 2. Experimental

### 2.1. Polymer Electrolyte Membrane Preparation

In this work, the entire chemical compounds were purchased from Sigma-Aldrich (Kuala Lumpur, Malaysia) and directly utilized to synthesize the polymer electrolytes. Initially, both CS (0.6 g) and DN (0.4 g) were dissolved in one percent of acetic acid (50 mL) at ambient temperatures for about 1.5 h. Then the CS and DN solutions were mixed homogeneously for about 2 h. To the CS,DN blend solution and NH_4_I salt (40 wt.%) was added with continuous stirring until the salt was dissolved completely. Next, into the solution, 10 mL of Zn(II)-complex was added.

Zn(II)-complex was prepared according to the procedure in the previous literature [21]. GL as a plasticizer was added to the system at different concentrations, as listed in Table 1. Next, the solution casting technique was employed to obtain a polymer electrolyte film. The thickness of the electrolyte was maintained within the range of 0.025–0.038 cm.

### 2.2. Electrode Preparation and EDLC Fabrication

A total of 3.25 g of dry activated carbon (AC) was blended with 0.25 g of carbon black by utilizing a planetary ball miller device for around 1 hr. Then, the dry mixture was added into the solution containing 0.5 g of polyvinylidene fluoride (PVdF) with 15 mL of *N*-methyl pyrrolidone (NMP). Later, a dense black solution was achieved after the solution mixture was mixed for few hours. Afterwards, the thick solution was varnished onto an aluminum foil by utilizing a doctor blade and was dried in an oven at 60 °C.

**Figure 1:**
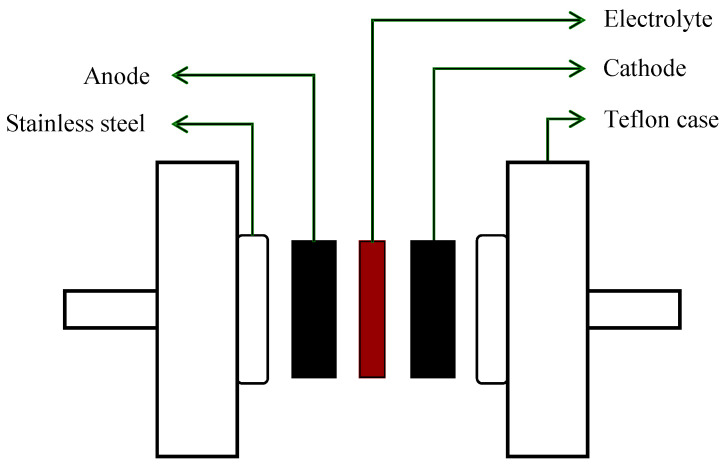
This electric double-layer capacitor (EDLC) diagram shows the highest conduction electrolyte sandwiched between two AC electrodes.

The EDLC was made of two AC electrodes, which have an area of 2.01 cm^2^ and the maximum conducting electrolyte that was pushed in a CR2032 coin cell (Kuala Lumpur, Malaysia). The outline of the AC electrodes and the maximum conducting electrolyte in the EDLC by using a CR2032 coin cell are illustrated in Figure 1.

### 2.3. Characterization Techniques

#### 2.3.1. Impedance, XRD and Morphology Analyses

The successfully prepared electrolytes were firstly tested using a LCR meter (HIOKI 3531-Z Hi tester, Nagano, Japan) to study their impedance properties. The measurement was conducted at room temperature within a frequency range of 50 Hz to 5 MHz.

X-ray diffractometer (Bruker AXS) (Malvern Panalytical Ltd., Malvern, UK) was used for the XRD acquisitions. The samples were exposed using a radiation source of a wavelength (λ = 1.5406 Å) and the scanning angle was measured over the range of 5–80° with a step size of 0.1°. The surface appearance of the electrolytes was investigated by FESEM (Hitachi SU8220 (FEI Quanta 200 FESEM, FEI Company, Hillsboro, OR, USA) at 500× magnification).

#### 2.3.2. Transference Number Analysis (TNM)

From the polarization of stainless steel (SS) | best conducting sample | SS, both an ionic (*t_i_*) transference number (TNM) and electronic (*t_e_*) were obtained. The working voltage to move electrons and ions was 0.20 V. The analysis was accomplished by means of V&A instrument (DP3003 digital DC power supply, Shanghai, China) at ambient temperature. The value of the *t_i_* was measured by the subsequent equations:

(1) $$t_{i}=\frac{I_{i}-I_{ss}}{I_{i}}$$

where *I_i_* and *I_ss_* are the initial and steady-state current, respectively, and the summation of *t_e_* and *t_i_* is equal to 1.

#### 2.3.3. Linear Sweep Voltammetry (LSV)

To make sure that the electrolyte was ready to be used in EDLC, the LSV method was implemented. It was noted that the cell arrangement was SS|. The arrangement was at its highest while conducting SPE|SS.A potentiostat (Digi-IVY DY2300, Shanghai, China) was used to learning the potential constancy of the electrolyte from 0 to 3.5 V at 100 mV/s.

#### 2.3.4. EDLC Characterization

Cyclic voltammetry (CV) was the first technique used for EDLC analysis. At various sweep rates, a Digi-IVY DY2300 potentiostat was used to accomplish the measurements.

The charge-discharge measurements were performed as the second test using a Neware battery cycler system (Shanghai, China) at the current density of 0.5 mA/cm^2^. Several significant parameters of the EDLC such as power density (*P*), specific capacitance (*C_s_*), energy (*E*) density, and equivalent series resistance (*ESR*) were measured by the following equations.

(2) $$C=\frac{i}{gm}$$

(3) $$ESR=\frac{V_{d}}{i_{}}$$

(4) $$E=\frac{1}{2}\left(CV^{2}\right)$$

(5) $$P=\frac{1}{4}\left(\frac{V^{2}}{mESR}\right)$$

where *g* stands for the slope of discharge part, *m* is the active material’s mass, *V_d_* is the potential drop, and *i* is the current applied.

## 3. Results and Discussion

### 3.1. Impedance Study

**Figure 2:**
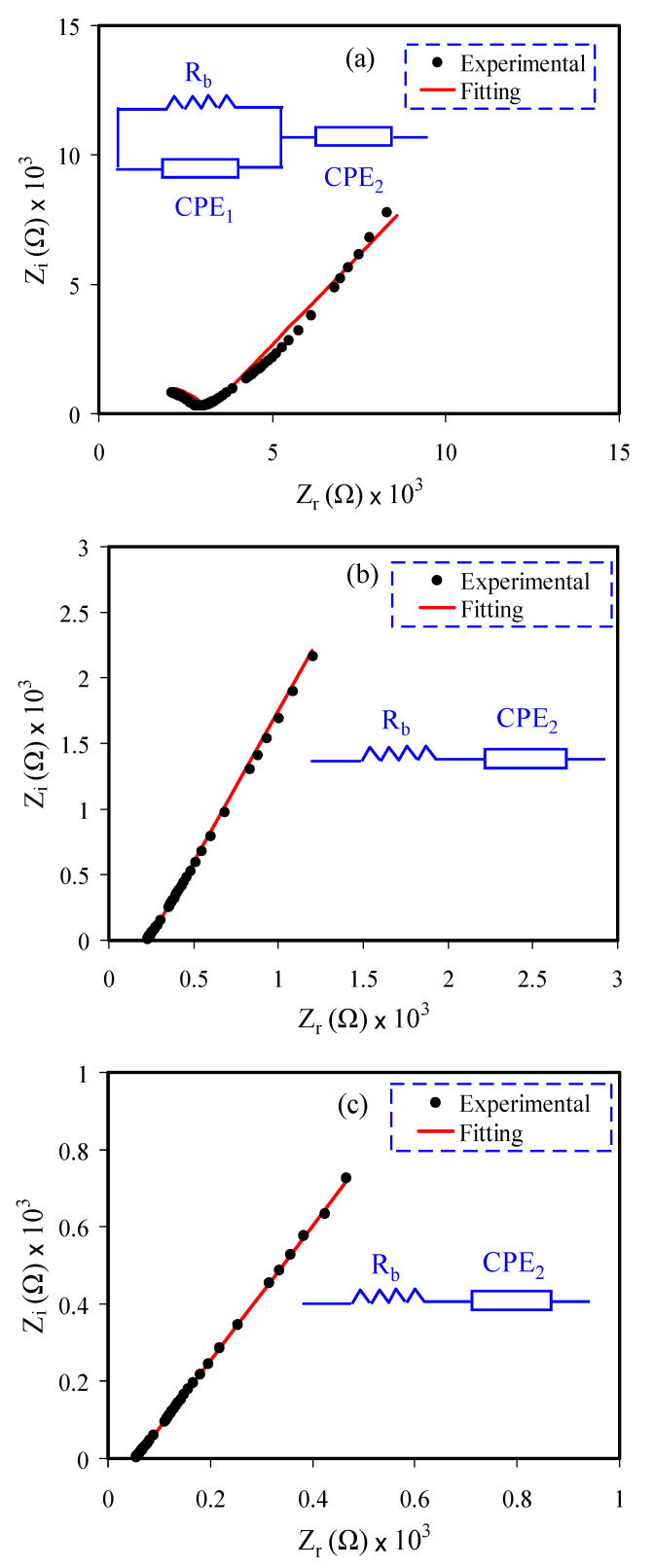
EIS plot for (**a**) CS DN1, (**b**) CSDN2, and (**c**) CSDN3 electrolytes with their equivalent circuit diagrams (insets) at room temperature in a frequency range of 50 Hz to 5 MHz.

Impedance spectroscopy is used as a well-organized practice to check the DC conductivity of polymer electrolytes. Ion-conducting electrolytes are a group of materials which obtained attention over recent years owing to their uses in a comprehensive number of electrochemical energy storage devices [29,30,31,32]. Complex impedance spectroscopy (CIS) is normally employed to separate a semicircle at a high frequency region, which corresponds to the conduction of ions in the bulk of the electrolytes and a spike at a low frequency region associated with the impact of electrode polarization [1,33]. To get more insights into ion migration in the current study, the electrical equivalent circuit (EEC) model has been studied. The EEC model is a simple method to indicate a picture for each system [34,35,36,37]. Figure 2a–c indicates the impedance plot with EEC model for each film. To achieve the overall picture for each system, the experimental EIS data was fitted with the EEC model. The Figure 2a–c insets indicate the equivalent circuits. Clearly, the semicircle emerged in the impedance plot of CSDN1 (Figure 2a); however, it is missed in the CSDN2 and CSDN3 systems (Figure 2b,c). At the low frequency region, the data points were due to the effect of electrode polarization, which was associated to the double layer capacitance creation between the electrodes and electrolytes. Through using the blocking electrodes in the EIS study, the electrolyte and electrode interfaces could be regarded as a capacitance [38]. For ideal capacitance, a vertical tail in the EIS plots should be seen at a low frequency region. Nevertheless, in this study, a tail angled with smaller than 90° was demonstrated rather than the vertical one. This is due to the double-layer capacitance located at the blocking electrodes or the toughness of the electrolytes and electrodes interfaces [39]. The determination of bulk resistance (*R*_b_) from the Nyquist plot for the electrolyte charge carriers and for two constant phase elements (CPEs) such as CPE_1_ and CPE_2_ are demonstrated in Figure 2.

The EEC is expressed by a parallel combination of *R*_b_ and CPE_1_ and it is in series with another CPE_2_ as the result of the tilted spike. The impedance of Z_CPE_ can be shown as [35,37]:

(6) $$Z_{CPE}=\frac{1}{C\omega^{p}}\left[cos\left(\frac{\pi p}{2}\right)-isin\left(\frac{\pi p}{2}\right)\right]$$

where *C*, *ω*, and *p* are the CPE capacitance, angular frequency, and *p* the deviation of the EIS plots from the imaginary axis in the EIS plots, respectively. Here, the *Z_r_* and *Z_i_* associated with the equivalent circuit (insets of Figure 2a) are shown as:

(7) $$Z_{r}=\frac{R_{b}{}^{2}C_{1}\omega^{p1}cos\left(\frac{\pi p_{1}}{2}\right)+R_{b}}{2R_{b}C_{1}\omega^{p1}cos\left(\frac{\pi p_{1}}{2}\right)+R_{b}{}^{2}C_{1}{}^{2}\omega^{2p1}+1}+\frac{cos\left(\frac{\pi p_{2}}{2}\right)}{C_{2}\omega^{p2}}$$

(8) $$Z_{i}=\frac{R_{b}{}^{2}C_{1}\omega^{p1}sin\left(\frac{\pi p_{1}}{2}\right)}{2R_{b}C_{1}\omega^{p1}cos\left(\frac{\pi p_{1}}{2}\right)+R_{b}{}^{2}C_{1}{}^{2}\omega^{2P1}+1}+\frac{sin\left(\frac{\pi p_{2}}{2}\right)}{C_{2}\omega^{p2}}.$$

The parameters *P_1_, P_2_, C_1_*, and *C_2_* are the capacitance of CPE_1_ at the bulk of the solution electrolyte, the capacitance of CPE_2_ inside the electrodes and electrolytes interfaces, nonconformity of the point from the original axis, and the semicircle from the imaginary axis, correspondingly.

*Z_r_* and *Z_i_* values of Z^*^ that are associated to the EEC (Figure 2b,c) insert) are shown below:

(9) $$Z_{r}=R+\frac{cos\left(\frac{\pi p_{2}}{2}\right)}{C_{2}\omega^{p2}}$$

(10) $$Z_{i}=\frac{sin\left(\frac{\pi p_{2}}{2}\right)}{C_{2}\omega^{p2}}.$$

The circuit element parameters for each sample are listed in Table 2. It is remarkable to see that the *R*_b_ decreased with increasing GL concentrations. At 42 wt.% GL concentration, more ions dissociated, thus the *R*_b_ decreased noticeably. Because of dissociation of more salts, the number of ions contributing to the conductivity increased.

When the *R*_b_ values and the film dimensions are considered, the equation below can be used to determine the DC conductivity:

(11) $$\sigma_{dc}=\left(\frac{1}{R_{b}}\right)\times \left(\frac{t}{A}\right)$$

where *t* and *A* are the film thickness and the stainless steel electrodes area. The DC ionic conductivity for each film is measured using the *R*_b_ and the film thickness and indicated in Table 3. It was noted that the highest plasticized film has the highest DC conductivity. The literature has reported that the DC conductivity in the range of 10^−5^ to 10^−3^ S/cm can properly be used in electrochemical energy storage applications [40,41,42,43]. Therefore, the highest conductivity film in the current study can be used in applications for ESDs.

### 3.2. XRD and Morphology Analyses

**Figure 3:**
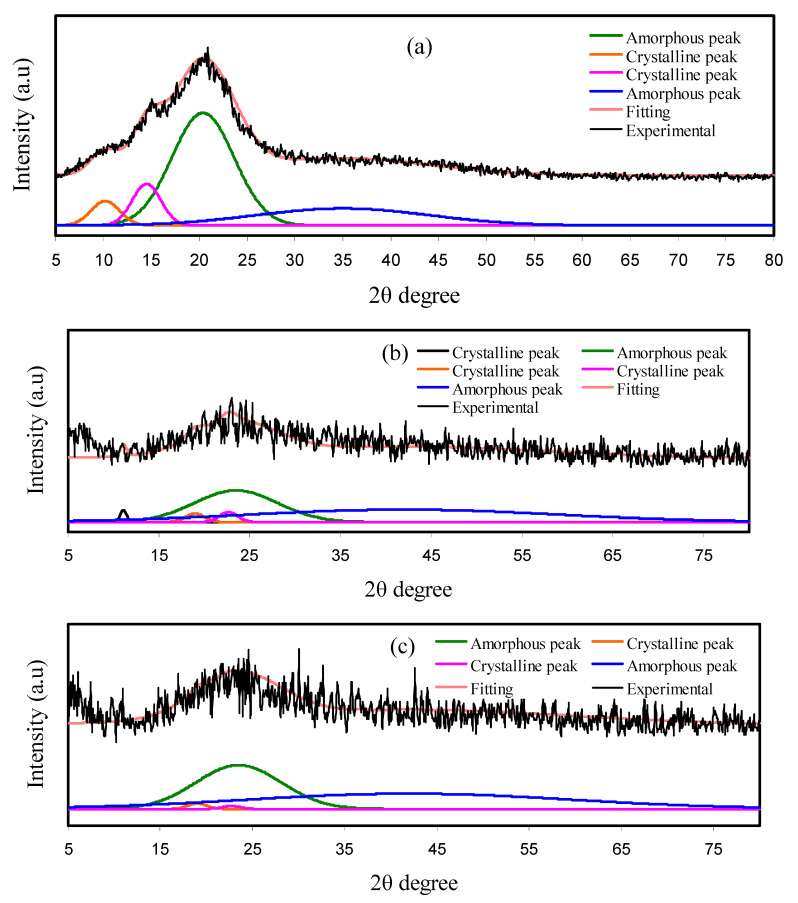
XRD pattern for (**a**) pure CS, (**b**) CSDN2, and (**c**) CSDN3 electrolytes over the range of 5–80° with a step size of 0.1°.

To explore the structural performance of the samples, the XRD patterns were acquired. The XRD patterns of pure CS and blend electrolyte films are shown in Figure 3a–c. Evidence revealed that pure CS is characterized by a semi-crystalline structure. From Figure 3a, it can be clearly noted that CS possesses a range of crystalline peaks. Regarding pure CS’s XRD pattern, two characteristic peaks at near 2θ° = 10.9°, 15°, and 20° demonstrate the crystalline part of the pure CS membrane’s average intermolecular distance [44,45,46]. CS’s rigid crystalline structure is primarily kept through hydrogen bonding (both intramolecular and intermolecular), which is the product of hydroxyl and amino groups by means of an absorbed water molecule [47,48,49]. Conversely, DN has two general broad peaks at 2θ =18 and 23° [50,51]. It is worth noting that the XRD pattern of the plasticized electrolyte system exhibits some broad peaks and also a few crystalline peaks can be seen. These wide hollows show the domination of the amorphous structure of the electrolyte system [50]. It is apparent that with the addition of 42 wt.% of GL the hollow intensity was considerably decreased, as exhibited in Figure 3c. This is a good indication of the decreasing of the crystalline structure of the electrolyte system and consequently improving the amorphous nature [52]. This result highlights that both peak intensity decreasing and peak broadening in XRD patterns are indicators of the amorphous structure’s dominance within the plasticized systems.

As a more interesting observation, upon the GL’s addition into the electrolyte system, the degree of crystallinity (*Xc*) decreased, which is associated to the complex creation among the polar groups of the polymers and the cations of the inorganic salts. Therefore, it decreases the crystalline structure within the electrolyte by destroying hydrogen bonding [53]. Hodge et al. reported that there is an association between *Xc* and peak intensity, in which the decrease in the intensity of the peak was considered as evidence of the amorphous feature of the film [54].

The crystalline and amorphous peaks were separated by the deconvolution method [55]. From the XRD deconvolution, the *Xc* of the diffraction peaks was obtained as illustrated in Figure 3a–c. It is noted that the sharp and narrow peaks represented the crystalline peaks, while the broad and large peaks are the amorphous peaks.

The GL addition of up to 42 wt.% creates greater amorphous peaks and smaller crystalline peaks, as shown in the CSDN3 system. The *Xc* for all films was obtained by Equation (12) and is listed in Table 4. The *Xc* of the pure CS is 15.97. The smallest degree of crystallinity, which is 1.69, is achieved for the CSDN3 system. This confirms that the CSDN3 system is the most amorphous system. The DC ionic conductivity values follow the trend of *Xc*

(12) $$Xc=\frac{A_{C}}{A_{T}}\times 100\%$$

where *A_c_* and *A_T_* are the area of total crystalline and the area of total amorphous and total crystalline, correspondingly. The Gaussian function mode in the OriginPro software was used to fit the XRD spectra.

**Figure 4:**
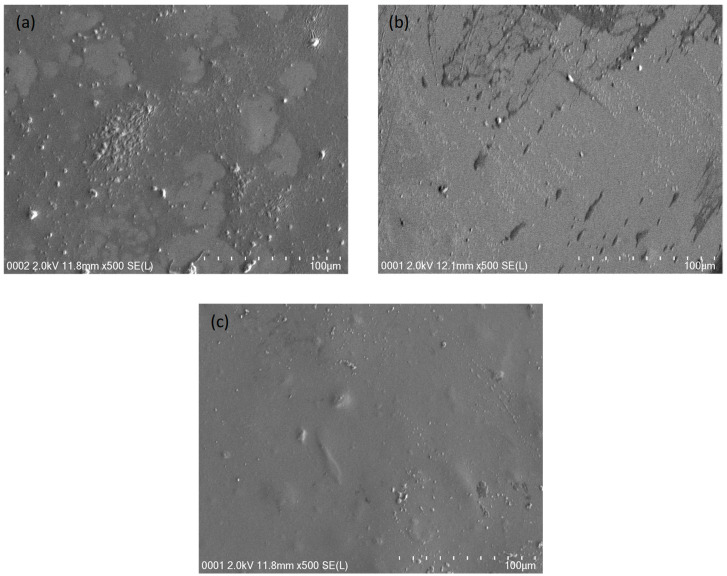
FESEM images for (**a**) CSDN1, (**b**) CSDN2, and (**c**) CSDN3 electrolytes at 500× magnification.

For each film, FESEM images were determined at 500× magnification in order to confirm the XRD and EIS results. The FESEM images are shown in Figure 4a–c. When the samples glycerolized by 14 and 28 wt.%, the surface of each film was emerged with few salts as shown in Figure 4a,b. As the amount of GL raised to 42 wt.% in the case of CSDN3, protrude salts were not clearly observed in the FESEM images. Consequently, a smooth surface morphology and uniform film with no phase segregation were achieved. It can be concluded that the FESEM images are supported with the XRD and the EIS results. It was revealed that the smooth morphology form can be linked to the amorphous structure of the electrolyte films. It was observed that, when the smooth surface electrolyte was obtained, the ions can be easily migrated; subsequently, the DC conductivity was improved [56].

### 3.3. Electrochemical Investigations

#### 3.3.1. TNM Analysis

**Figure 5:**
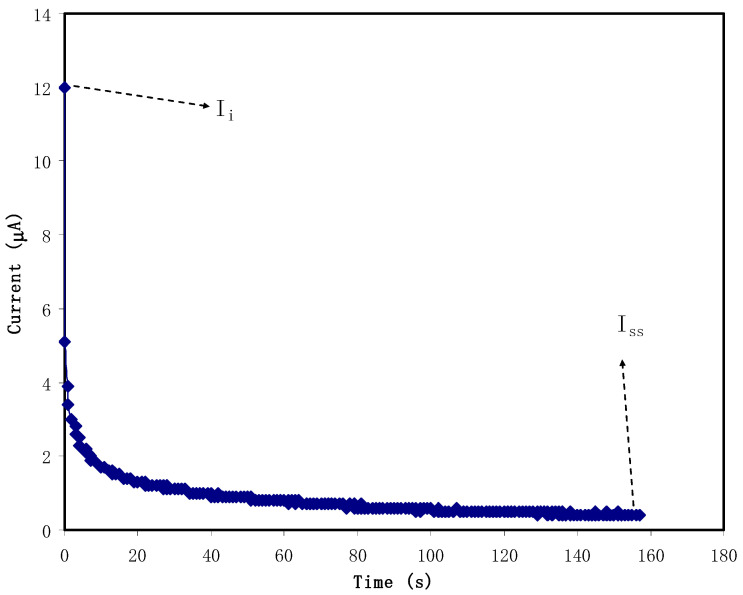
The plot of a polarized cell at ambient temperature with a working voltage of 0.2 V.

In order to identify the main charge carrier in the electrolyte system, TNM analysis was utilized. Once the system subjected by 0.20 V, the current begins to drop from 12 μA until the electrolyte system reaches saturation. The polarization of current versus time for the cell is portrayed in Figure 5. At first, cations, anions, and electrons are conducted in the electrolyte, which explains the high current (*I_total_* = *I_i_* + *I_e_*) at the initial stage. The current drops to ~1 μA after 30 s. At this time, some ions are polarized and the current is affected by the flow of electrons [57]. A complete polarization process is achieved when the current stabilized at 0.2 μA. The values of *t_e_* and *t_i_* are found to be 0.017 and 0.983, respectively. The *t_i_* is near the ideal value. Therefore, it was revealed that the ions are responsible for the conduction process within the CS:DN:NH_4_I:Zn(II)-complex:GL electrolyte system.

A value of 0.966 was reported in a system of Zn(II)-complex, CS, ammonium fluoride (NH_4_F), and GL [20]. The presence of high dielectric plasticizer also can be the reason of high *t_i_*. GL has a dielectric constant of 42.5, which weakens the bond of NH_4_I and produces more free ions e.g., NH_4_^+^, H^+^, and I^−^ to produce a charge double-layer allocated at the surface of the electrodes [58].

#### 3.3.2. LSV Study

**Figure 6:**
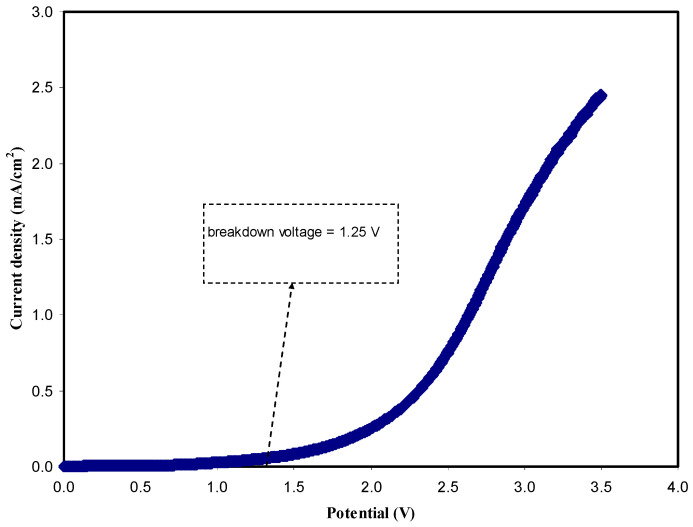
LSV plot of the best conducting sample (CSDN3) from 0 to 3.5 V at a sweep rate of 100 mV/s.

The electrochemical stability and working potential range of the polymer electrolyte is an important criterion due to high performance of energy devices. LSV was carried out to observe the electrochemical stability of the highest conducting CS:DN:NH_4_I:Zn(II)-complex:GL system. Figure 6 shows the LSV plot of the best conducting CS:DN:NH_4_I:Zn(II)-complex:GL system at room temperature. No obvious current was observed in the range 0–1.25 V. As the potential goes beyond 1.25 V, the current was recorded to increase gradually. The increase in current indicates the starting of the redox process [59]. At this point, the electrolyte started to decompose and could no longer perform properly. Commonly, the average electrochemical window is ~1 V for proton-based energy storage devices [60]. Thus, the best conducting sample for a CS:DN:NH_4_I:Zn(II)-complex:GL system can be used to conduct ions for the application of EDLC.

#### 3.3.3. CV Analysis

**Figure 7:**
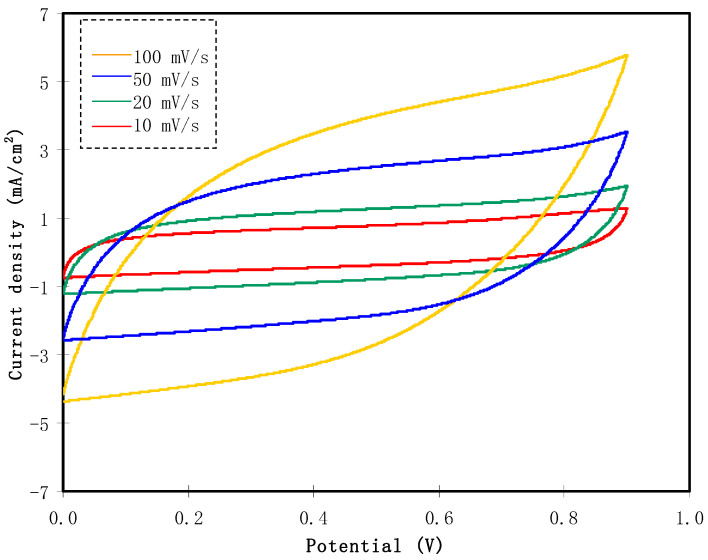
CV plot of the EDLC at current density of 0.5 mA/cm^2^ and potential range of 0 to 0.9 V for various sweep scan rates.

Typical EDLCs undergo a non-Faradaic mechanism for the energy storing process when non redox reactions happen. This condition was checked using cyclic voltammetry (CV) analysis at various sweep rates (*a*) to see its effect on the specific capacitance (*C_cyc_*), which can be obtained using the equation below:

(13) $$C_{cyc}=\int_{V_{i}}^{V_{f}}\frac{I\left(V\right)dV}{2ma\left(V_{f}-V_{i}\right)}$$

where *I(V)dV* is the area of the CV plot in Figure 7 using integration function via Origin 9.0 software. *V_f_* and *V_i_* are 0.9 V and 0 V, respectively. The values of *C_cv_* obtained for 10, 20, 50, and 100 mV/s are 20.5, 28.5, 37.5, and 60.0 F/g, respectively. It is obvious from these findings that the specific capacitance is dependent on sweep rates. At large sweep rates e.g., 100 and 50 mV/s, the flow of cations and anions in the electrolyte is unstable, thus providing improper charge double-layer development at the surface of AC electrodes. This situation also creates current dependent potential, which clarify the shape of leaves for large sweep rates [61]. At a slower sweep rate, suitable polarization progression can be done. This condition leads to the current’s independent potential and creates a rectangular shape CV plot [62]. A charge double-layer is produced when the ions from electrolytes and electrons from electrodes face attractive and repulsive forces. As a result, within the CV plot, no redox peaks were observed. It was observed that the activated carbon|CS:DN:NH_4_I:Zn(II)-complex:GL activated carbon EDLC in this study has a capacitive behavior.

#### 3.3.4. Galvanostatic Charge-Discharge (GCD) Analysis

**Figure 8:**
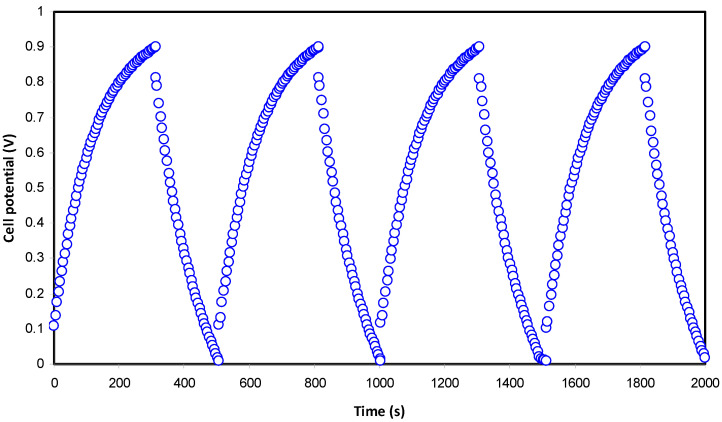
Typical charge-discharge plot of the fabricated EDLC at selected cycles.

**Figure 9:**
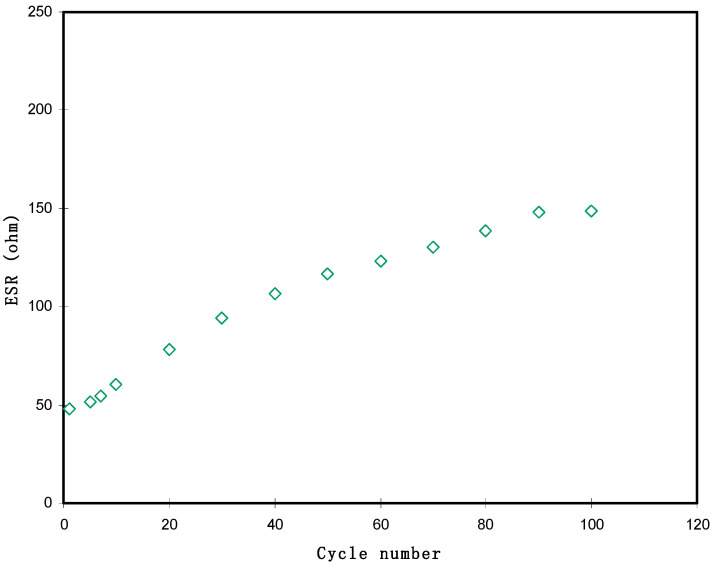
*ESR* pattern of the fabricated EDLC throughout the 100 cycles.

The cyclic durability of the EDLC and the discharge/charge mechanism can be discussed more through charging and discharging processes. Figure 8 exhibits the GCD profiles of the EDLC at the selected cycles. Normally, the slope of the discharge part will be non-linear for batteries or a redox-capacitor; unlike for batteries, EDLC has a linear slope [63]. According to the Figure 8, it can be seen that when the potential dropped sharply, the slope of the discharge section is virtually lined. The value of drop voltage (*V*_d_) ranged from 0.0478 to 0.1488 V throughout the 100 cycles. As we obtained *V*_d_, we could calculate the value of *ESR*, which can be seen in Figure 9. The *ESR* of the EDLC at the 1st cycle was 47.8 ohm. It is noticeable that the slope of *ESR* increment was higher after the 30th cycle. Typically, in EDLC, ions are trying to recognize the pattern of conduction and some of the ions recombined, thus increasing the *ESR*. However, beyond the 30th cycles, the value of *ESR* started to stabilize. Hence, other parameters are expected to be consistent with the buildup of *ESR* in this EDLC.

**Figure 10:**
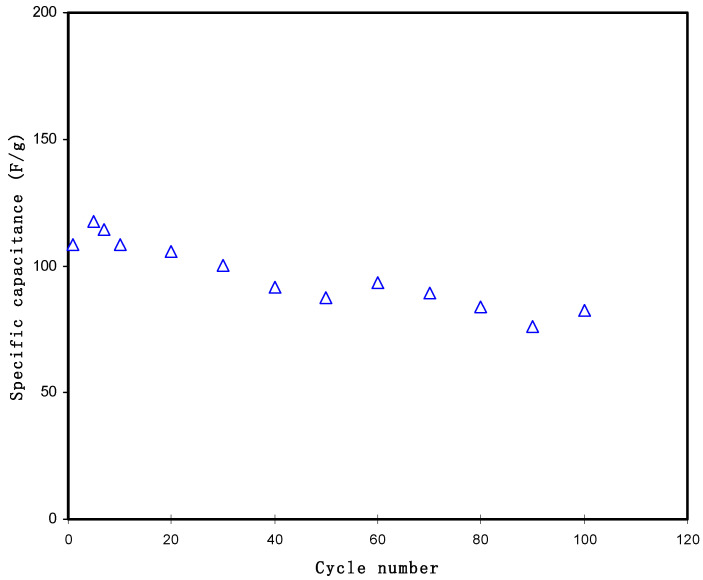
Specific capacitance versus cycle numbers for the EDLC over 100 cycles.

The calculated *C_s_* value of the EDLC for 100 cycles is shown in Figure 10. The *C_s_* at the 1st was identified to be 108.3 F/g. The *C_s_* increased to 117.5 F/g at the 5th cycle and continued to decrease to be almost constant at an average value of 90.4 F/g up to the 100th cycle. The pattern of *C_s_* in Figure 10 is the opposite of *ESR* in Figure 9, where the increment of internal resistance at cycle numbers below 30 is the reason for the *C_s_* decrement. The *C_s_* obtained is significant when matched to the low *C_s_* values of 1.7–2.1 F/g and 2.6–3.0 reported for Li- and Mg-based PEO EDLC incorporated with ionic liquids [61]. Tripathi et al. [64] reported a maximum *C_s_* of 61.7 F/g for EDLC with the electrolyte system of PVdF-BMIMCl-TEABF_4_-EC-PC. The specific capacitance in this study is quite close to those of gel polymers electrolytes-based EDLC developed by Boonen et al. [65] and Łatoszynska et al. [66], which were 87.3 F/g and 90 F/g, separately. Thus, a polymer blend host can be considered as a novel technique to prepare SCs with good specific capacitance at room temperature.

**Figure 11:**
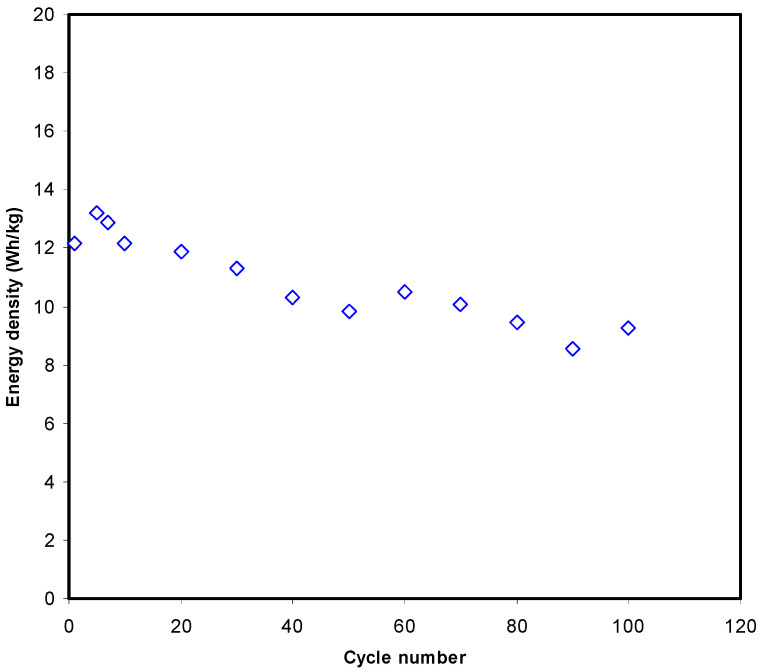
The plot of *E* of the fabricated EDLC over 100cycles.

**Figure 12:**
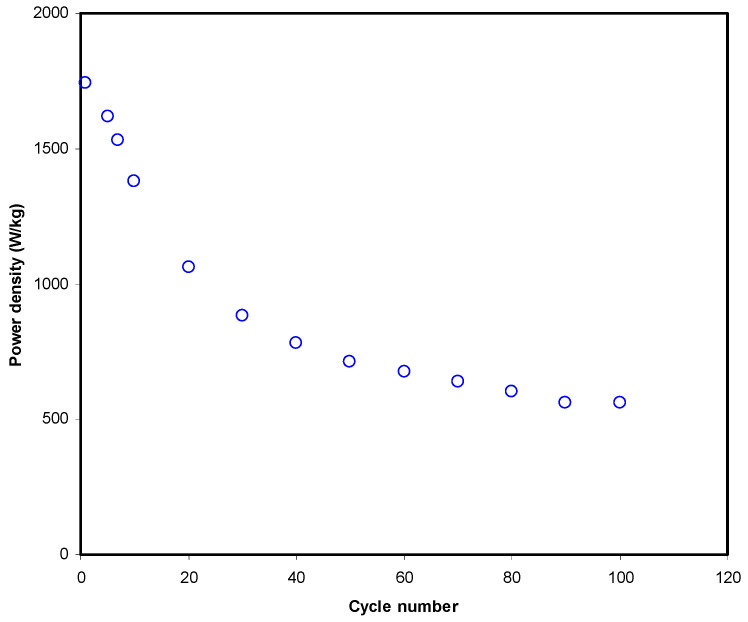
Power density trend of the fabricated EDLC over 100 cycles.

Figure 11 shows that energy density (*E*) at the 1st cycle is 12.2 Wh/kg. Then, *E* decreased to 11.3 Wh/kg and stabilized at an average of 9.72 Wh/kg from the 40th cycle to the 100th cycle. Hence, it can be assumed that beyond 40th cycle the conduction of ion has almost the same energy barrier [34]. The *E* in this work is comparable to other EDLC reported using various materials. Hina et al. [67] reported that the value of *E* for their EDLC is in the range of 9.82 to 21.6 Wh/kg depending on the concentration of lithium triflate (LiTf). EDLC by Mazuki et al. [68] achieved 1.19 Wh/kg for a CMC-PVA-NH_4_Br system. The high *E* could be due to the presence of plasticizer in this work. The outcomes of this work reveal that biopolymer-based electrolytes are important for energy storage applications. Moreover, the power density (*P*) values are calculated if *ESR* values are known, which can be seen in Figure 12. The *P* value at the 1st cycle is determined to be 1743.4 W/kg and it drops to 560 W/kg as the EDLC finished 100 cycles. The pattern of *P* is in good agreement with the *ESR* trend. This is due to the degradation of electrolytes that happens when the resistance raises, causing ionic aggregation from the rapid process of charging/discharging, thus causing a decreased *P* at a high cycle number [69]. The values of *E* and *P* are obviously reliant on the active material’s mass loading in the fabrication of EDLC. The quite low current and small mass loading is documented to be responsible for producing an improved electrochemical performance [70]. Remarkably, the current biodegradable polymer based system has shown a clear improvement in the properties of the prepared electrolyte with enhanced EDLC performance compared to the other polymer based electrolytes from the literature, as presented in Table 5.

## 4. Conclusion

In summary, a solution casting scheme was used to produce plasticized H^+^ conducting composite electrolytes. The conductivity of the resulting electrolyte was enhanced by adding 42 wt.% GL to deliver the conductivity of 3.44 × 10^−4^ S cm^−1^. From the XRD analysis, it was demonstrated that the maximum amount of plasticized system offers the lowest *X_c_*, and the result was calculated to be around 1.69.

In addition, the XRD measurements was connected with the *X_c_* trend and the DC ionic conductivity of the polymer electrolyte. From the FESEM images, it was observed that, at the maximum amount of GL concentration, the film exhibited a homogenous layer and smooth surface morphology. The high value of *t_i_* (0.983), ions had been considered to be the primary contributor in the ionic transport process in the polymer electrolytes. The highest conducting system has been determined to be stable at up to 1.25 V from LSV analysis. This threshold potential supported the possibility of using the electrolyte in energy storage devices. No redox peaks emerged in the CV plot of the EDLC, which signifies that the anodic and cathodic reactions are absent at the activated carbon electrode’s surface. The specific capacitance of the EDLC was found to be 108.3 F/g at the 1st cycle with internal resistance ranging from 47.8 ohm to 148.4 ohm. The energy and power density at the 1st cycle were found to be 12.2 W/kg and 1743.4 W/kg, respectively, despite the significant improvement in the properties of these biodegradable polymer electrolytes reported in the literature. Nevertheless, intensive study is still needed to enhance the overall performance of this natural polymer electrolyte to obtain sufficient ionic conductivity, electrochemical stability, and a good compatible electrolyte/electrode interface for energy device applications. Choosing appropriate polymers for blending with embedding suitable fillers and plasticizers could be among the most effective approaches. 

## Figures and Tables

**Table 1:** Designation of the polymer electrolytes.

GL (wt.%)	Sample Code
12	CSDN1
28	CSDN2
42	CSDN3

**Table 2:** The EEC fitting parameters for each plasticized system at room temperature.

Sample	n_1_ (rad)	n_2_ (rad)	K_1_ (F^−1^)	K_2_ (F^−1^)	C_1_ (F)	C_2_ (F)
CSDN1	0.773	0.604	1.0 × 10^9^	3.05 × 10^5^	1.0 × 10^−9^	3.28 × 10^−6^
CSDN2	-	0.738	-	1.68 × 10^5^	-	5.95 × 10^−6^
CSDN3	-	0.668	-	3.9 × 10^4^	-	2.56 × 10^−5^

**Table 3:** DC ionic conductivity (σ_dc_) of CS:DN:NH_4_I:Zn(II)-complex:GL system at room temperature.

Code	Ionic Conductivity (σ_DC_) (S·cm^−1^)
CSDN1	4.06 × 10^−6^
CSDN2	6.43 × 10^−5^
CSDN3	3.44 × 10^−4^

**Table 4:** The *Xc* for each film calculated from a deconvoluted XRD pattern.

Electrolyte	Degree of Crystallinity (%)
Pure CS	15.97
CSDN2	5.82
CSDN3	1.69

**Table 5:** Comparison between the performance of the prepared polymer electrolyte with other polymer based electrolytes in terms of DC conductivity ($\sigma_{DC}$) specific capacitance (*C_s_*), energy density (E).

Electrolyte Composition	*σ_DC_* (S cm^−1^)	*C_s_* (F g^−1^)	E (Wh kg^−1^)	Ref.
Dextran:NH_4_Br	(1.67 ± 0.36) × 10^−6^	2.05	-	[71]
PVA:Dextran:NH_4_I	2.08 × 10^−5^	4.2	0.55	[72]
MC-NH_4_NO_3_- PEG	2.1 × 10^−6^	38	3.9	[73]
CS-PVA-Mg(CF_3_SO_3_)_2_:GL	1.016 × 10^−5^	32.69	-	[74]
Corn starch: LiClO_4_: SiO_2_	1.23 × 10^−4^	9.83	0.9	[75]
PVA:LiClO_4_:TiO_2_	1.3 × 10^−4^	12.5	1.56	[76]
**CS:DN:NH_4_I:Zn(II)-complex:GL**	**3.44 × 10^−4^**	**108.3**	**12.2**	**This work**

where: NH_4_Br = Ammonium bromide, MC = Methylcellulose, NH_4_NO_3_ = Ammonium nitrate, PEG = Poly(ethylene glycol), Mg(CF_3_SO_3_)_2_ = Magnesium triflate, LiClO_4_ = Lithium perchlorate, SiO_2_ = Silicon dioxide, TiO_2_ = Titanium dioxide.

## Data Availability

No new data were created or analyzed in this study. Data sharing is not applicable to this article.
